# Postural Adjustments and Biomechanics During Gait Initiation and Obstacle Negotiation: A Comparison Between Akinetic-Rigid and Hyperkinetic Parkinson’s Disease

**DOI:** 10.3389/fphys.2021.723628

**Published:** 2021-11-04

**Authors:** Marcela Zimmermann Casal, Leonardo Alexandre Peyré-Tartaruga, Ana Paula Janner Zanardi, André Ivaniski-Mello, Lucas de Liz Alves, Aline Nogueira Haas, Flávia Gomes Martinez

**Affiliations:** ^1^Exercise Research Laboratory, Universidade Federal do Rio Grande do Sul (UFRGS), Porto Alegre, Brazil; ^2^Physical Therapy College, Centro Universitário de Cascavel (UNIVEL), Cascavel, Brazil

**Keywords:** Parkinsonian disorders, postural adjustments, kinematic, locomotion, stiffness, electromyography

## Abstract

**Background:** Individuals with Parkinson’s disease (PD) exhibit different combinations of motor symptoms. The most frequent subtypes are akinetic-rigid (AK-R) and hyperkinetic (HYP). Motor symptoms, such as rigidity and bradykinesia, can directly affect postural adjustments and performance in daily tasks, like gait initiation and obstacles negotiation, increasing the risk of falls and functional dependence.

**Objective:** To compare postural adjustments and biomechanical parameters during the gait initiation and obstacle negotiation of people with AK-R and HYP PD and correlate with functional mobility and risk of falls.

**Methods:** Cross-sectional study. Thirty-three volunteers with PD were divided into two groups according to clinical motor manifestations: AK-R (*n* = 16) and HYP (*n* = 17). We assessed the anticipatory (APA), compensatory (CPA) postural adjustments analyzing kinematic, kinetic and, electromyographic parameters during the gait initiation and obstacle negotiation tests. We applied independent *T*-tests and Pearson correlation tests for comparisons and correlations, respectively (α = 0.05).

**Results:** In the APA phase of the gait initiation test, compared to the functional HYP group, the AK-R group showed shorter time for single support (*p* = 0.01), longer time for double support (*p* = 0.01) accompanied by a smaller first step (size, *p* = 0.05; height, *p* = 0.04), and reduced muscle activation of obliquus internus (*p* = 0.02). Similarly, during the first step in the obstacle negotiation test, the AK-R group showed less step height (*p* = 0.01) and hip excursion (*p* = 0.02), accompanied by a reduced mediolateral displacement of the center of pressure (*p* = 0.02) during APA, and activation of the gluteus medius (*p* = 0.02) and the anterior tibialis (*p* = 0.04) during CPA in comparison with HYP group.

**Conclusion:** The findings suggest that people with AK-R present impaired postural adjustments during gait initiation and obstacles negotiation compared to hyperkinetic PD. Based on defined motor symptoms, the proposition presented here revealed consistent postural adjustments during complex tasks and, therefore, may offer new insights onto PD motor evaluation and neurorehabilitation.

## Introduction

Parkinson’s disease (PD) is a chronic and progressive disease of the central nervous system that affects motor behavior. Bradykinesia, akinesia, tremors, postural changes, freezing, axial, and intersegmental rigidity are characteristic symptoms in PD (Lees et al., 2009; Peterson and Horak, 2016), but this disorder has a variable clinical manifestation and heterogeneity concerning the progression of symptoms (Jankovic et al., 1990; Van Rooden et al., 2011). Commonly, these symptoms are divided into akinetic-rigid (AK-R) and hyperkinetic tremor-dominant (HYP) PD (Eggers et al., 2011; Zhang et al., 2015; León-Jiménez, 2019). Evidence reveals that PD subtypes have a different clinical course and prognosis (Marras et al., 2002; Rajput et al., 2009).

Postural control during dynamic activities requires the integration of multiple sensory and motor pathways so that the central nervous system can coordinate the anticipatory/compensatory (postural) and intentional (movement) components (Chang et al., 2004; Hass et al., 2005). PD patients perform complex tasks as gait initiation and obstacle negotiation, presenting reduced anticipatory and compensatory postural adjustments (APA and CPA, respectively) magnitudes compared to healthy individuals (Latash et al., 1995; Mancini et al., 2009; Yelshyna et al., 2016; Schlenstedt et al., 2018). Specifically, people with PD tend to have lower magnitudes of postural adjustments, hypometric APA, shorter stride length, and vertical oscillation (Vitório et al., 2010; Plate et al., 2016; Bonora et al., 2017; Schlenstedt et al., 2018).

Parkinson’s disease patients perform gait initiation poorly (Gantchev et al., 1996; Halliday et al., 1998; Hass et al., 2008; Delval et al., 2014). Abnormalities occur for this population during the APA and CPA phases (Dibble et al., 2004; Carpinella et al., 2007; Hass et al., 2008; Jacobs et al., 2009). Notably, they extend the duration of the postural phase (Gantchev et al., 1996; Rosin et al., 1997; Halliday et al., 1998) and reduce the center of pressure (COP) oscillation (Dibble et al., 2004; Morris et al., 2005; Carpinella et al., 2007; Hass et al., 2008) as the motor symptoms progress. The first step or gait initiation has been specially investigated due to its relationship with the major functional impact of this disease, considering frequent symptoms such as freezing and festination during the attempt to start the locomotion (Hass et al., 2005; Rocchi et al., 2012; Roemmich et al., 2012). Likewise, obstacle negotiation is an important skill related to daily tasks, and its poor execution can be linked to an increased risk of falls (Vitório et al., 2010; Conceição et al., 2019).

The literature suggests that PD subtypes have a different clinical course and prognosis (Marras et al., 2002; Rajput et al., 2009) and should be considered in clinical practice. However, no study explored these groups concerning motor behavior and locomotion. Likewise, although some studies have investigated postural automatisms during daily motor tasks in PD (Latash et al., 1995; Mancini et al., 2009; Schlenstedt et al., 2018), none assessed APA and CPA comparing PD subtypes. Thus, the innovative objective of this study was to analyze and compare postural adjustments and biomechanical parameters related to the beginning of gait and negotiation of obstacles in people with AK-R and HYP DP. Our hypothesis is that people with AK-R PD would have greater impairment of APA and CPA, reflected in less COP displacement and less electromyographic activity from the stabilizer muscles, as well as lower values for step size, step height, and range of hip motion during execution tasks.

## Materials and Methods

### Participants and Ethics Statement

This study is a cross-sectional study. We included people of any gender, over the age of 50, diagnosed with idiopathic PD, 1 to 3 on the Hoehn and Yahr (1967) scale. They should be on regular drug treatment and have the ability to understand verbal instructions to perform tests. Also, they did not participate in any exercise program in the last 3 months. We determined the following exclusion criteria: having cognitive impairment, with Montreal Cognitive Assessment reaching at least 21 points (Tumas et al., 2016), deep brain stimulation surgery, history of vertigo, surgeries in lower limbs during the last year, use of prostheses in the lower limbs, severe heart diseases or other associated neurological diseases and not having conditions of ambulation. The Research Ethics Committee involving Human Beings (CAAE number: 69919017.3.0000.5347 of the *Universidade Federal do Rio Grande do Sul*, Brazil) approved this study. The procedures conformed to the latest revision of the Declaration of Helsinki. Before signing the informed consent form, all participants were aware of the potential risks and discomforts associated with this study.

### Assessment Tools

We used an anamnesis form to collect personal information, history of PD, main complaints and symptoms, health history, lifestyle, and physical activity, and we used an evaluation form to collect anthropometric data. The Unified Parkinson’s Disease Rating Scale (UPDRS-III) was also used to assess the predominance of the motor symptoms, dividing the sample into two groups: AK-R and HYP. For that, we calculated a “HYP score” and a “AK-R score” for each patient: the HYP score was derived from the items 20 (tremor at rest) and 21 (action or postural tremor of hands) divided by 7 (the number of single sub items included). The AK-R score was derived from the items 18 (speech), 19 (facial expression), 22 (rigidity), 27 (arising from chair), 28 (posture), 29 (gait), 30 (postural stability), and 31 (body bradykinesia and hypokinesia) divided by 12 (the number of single sub items included). The patient was classified as HYP type if the score was at least twice the AK-R score. The patient was classified as AK-R type if the score was at least twice the HYP score (Eggers et al., 2011; Lewis et al., 2011). Moreover, cognitive function was determined using the Montreal cognitive assessment. The Hoehn and Yahr scale is used to classify PD signs and symptoms (Hoehn and Yahr, 1967; Scalzo et al., 2009) and falls efficacy scale-international (FES-I) questionnaire was used to assess fear of falling (Yardley et al., 2005).

The individuals performed the 10-m walk test to determine the self-selected walking speed (SSS) and the timed up and go test to measure functional mobility (Morris et al., 2001). To eliminate the acceleration and deceleration component, they were asked to start walking 2 m before beginning the course and finishing 2 m after the 10 m course (Watson, 2002).

Electromyographic data (EMG) were collected using three electromyographs (Miotool 400, Miotec), from four channels each, with a sampling frequency of 2000 Hz per channel, gain variation of 200 to 1000 times and common rejection mode greater than 126 dB. Surface adhesive electrodes of bipolar configuration, model Mini Medi-trace 100, of the Kendall brand, with 10 mm of conductive area radius and 15 mm of total radius were used. Skin preparation and electrode placement followed the recommendations of the surface electromyography for the non-invasive assessment of muscles (SENIAM, Merletti et al., 2001).

We used a 3D force platform (AMTI, OR6-6, Watertown, MA, United States) coupled to a wooden walkway with a non-slip surface approximately 3 m long to acquire kinetic data. The sampling frequency was 100 Hz.

Kinematic data were collected using a 3D motion capture system (Vicon, Oxford, United Kingdom), using six Bonita 10^®^ infrared cameras, with a sampling frequency of 100 Hz and 1MP resolution. Thirty-six reflective markers were positioned bilaterally on the participants’ head, shoulders, torso, arm, pelvis, legs, and feet, based on the standard 15-segment biomechanical model previously established in the Gait-Vicon-Fullbody Plug-in, which calculates the joint kinematics from the spatial orientations of the markers (X, Y, and Z coordinates) and the anthropometric measurements of the individual.

### Data Collect

All participants attended two times to collect data. All evaluations were performed during the “on period” of PD medication, up to 3 h after ingestion. On the first day, we analyzed the previous evaluation to verify the fulfillment of the eligibility criteria based on the anamnesis form. After this stage, the UPDRS - III and Hoehn and Yahr scales were applied. The kinetic classification (akinetic-rigid or hyperkinetic) of each participant was determined based on the UPDRS-III domains (Eggers et al., 2011; Lewis et al., 2011). Then, the patients answered the FES-I and performed the 10-m walk test and the timed up and go test. The participants were familiarized with the gait initiation and obstacle negotiation tests.

On the second day, we performed the procedures for collecting the main data of the study. Initially, the anthropometric data necessary for the collection of kinematic data were measured: body mass (kg), height (cm), length of the lower limbs (mm), the distance between the femoral condyles (mm), distance between the malleoli (mm), distance between the epicondyles (mm), and distance from the tubercle of the scaphoid bone to the pisiform bone (mm). Then the participant was directed to the preparation process for the collection of electromyographic data.

The participant remained lying comfortably on a stretcher, while the researcher underwent a skin preparation procedure on the investigated muscles (trichotomy, abrasion, and cleaning) and pairs of surface electrodes were placed on the belly of the erector spinae longissimus (EEL), obliquus internus (OI), gluteus medius (GLM), rectus femoris (RF), biceps femoris (BF), anterior tibialis (TA), and gastrocnemius medialis (GAM) muscles of the most affected side. In addition, reference electrodes were placed on the individuals’ medial malleoli. Reflective markers were also placed at pre-determined anatomical references necessary for the analysis of movement through the VICON motion capture system.

Then, the participants were positioned on the force platform, barefoot, with their feet at a comfortable distance, no greater than the distance between the acetabulae, and were instructed to remain in orthostasis with their eyes open to collect the parameters in a situation of rest, prior to the stimulus of imbalance. After, the gait initiation and obstacle negotiation tests were performed, described below, starting from the force platform, when the acquisition of kinetic data in sync with EMG and kinematic data was performed.

#### Gait Initiation

The participant was instructed to remain static on the force platform and, then start the gait movement, walking through a self-selected walkway.

#### Obstacle Negotiation

The participants started again on the force platform and started to walk over an obstacle 10 cm high.

An electronic device synchronized the data acquisition systems since the collection involves independent instruments with simultaneous measurement. EMG data were collected from the most affected side during the first step with the contralateral leg, thus evaluating the supporting limb.

### Data Processing

Electromyographic data were processed and analyzed using a routine in the MatLab software (The MathWorks^®^, Natick, MA, United States). For the calculation of the integrals of the EMG (∫EMG), the raw data were filtered with Butterworth bandpass (40–450 Hz, second order) and low pass (40 Hz, second order) filters (Krishnan et al., 2012), rectified with full-wave rectification. The postural automatisms were analyzed through the ∫EMG in the APA period (−100 ms to +50 ms) and the CPA (+50 ms to +200 ms) – in relation to T0 – of the muscles in the situations: gait initiation and obstacle negotiation. T0 was considered the beginning of the lower limb movement for both tasks. The base activity of each muscle was calculated from the ∫EMG in the period from −550 ms to −400 ms in relation to t0 (Santos et al., 2010).

The kinetic variables were evaluated by means of the vertical force peak and the displacement of the anteroposterior and mediolateral COP during APA and CPA periods. The signals were acquired at 100 Hz and were filtered using the second order Butterworth low pass filter, with a cutoff frequency of 10 Hz. COP toward their anteroposterior (COPy) and mediolateral (COPx) components were calculated (Esposti et al., 2013).

VICON NEXUS^®^ 1.8 software was used to acquire and reconstruct the kinematic data. The angular variables were determined by calculating the range of motion (maximum value minus minimum value) of the hip, knee and ankle in the first step in each test. The kinetic and kinematic data were determined by a mathematical routine built using the Labview software (National Instruments 8.5, Austin, United States), where the size (anteroposterior length), height (vertical oscillation) and width (mediolateral length) of the first step were calculated, in addition to the single and double support time. A third order Butterworth digital filter was applied, low pass and the cutoff frequency was defined by residual analysis (Winter, 2005).

**FIGURE 1:**
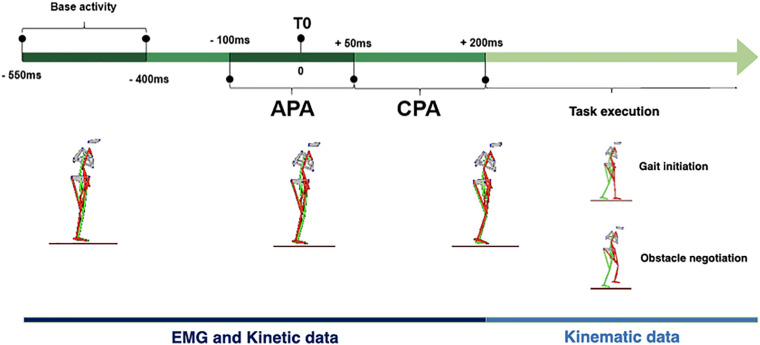
Timeline outlining course (left to right) of the gait initiation and obstacle negotiation tests: base activity, anticipatory postural adjustment (APA), start of movement during the APA (T0), compensatory postural adjustment (CPA), and task execution periods.

The summary of data processing for all study variables is described in Table 1. The APA, CPA, and execution periods, as well as the analyzes carried out in each stage, are shown in Figure 1. The dataset is available in doi: 10.6084/m9.figshare.14759037.

**TABLE 1:** Summary of data processing for study variables.

**Variables**	**Processing**
APA electromyographic activity (μV)	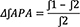
CPA electromyographic activity (μV)	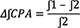
Anteroposterior COP displacement (cm)	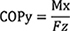
Mediolateral COP displacement (cm)	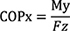
Vertical force peak (% Body Weight)	Maximum value of vertical force in APA periods
Step size (cm)	Anteroposterior distance from the center of mass during the first step
Step height (cm)	Maximum minus minimum segmental vertical distance of the feet during the first step
Step width (cm)	Maximum segmental mediolateral distance of the feet during the first step
Stride time (s)	Time of the first stride
Single support time (s)	Stride period when one foot is in contact with the ground
Double support time (s)	Stride period when both feet are in contact with the ground
Range of motion of the hip, knee, and ankle (degrees)	Maximum minus minimum value of hip, knee and ankle in first step

*APA, anticipatory postural adjustment; CPA, compensatory postural adjustment; μV, microvolts; ∫, integral activity; COP, center of pressure; Mx, moment on the mediolateral axis; My, moment on the anteroposterior axis; Fz, vertical ground reaction force.*

### Statistical Treatment

Data is presented in descriptive measures, using means, standard deviations and standard error for continuous measurements. Categorical data are presented as frequencies and relative percentages. Data normality and homogeneity were verified using the Shapiro-Wilk and Levene tests, respectively. For comparison between groups, the Mann-Whitney *U* test for non-parametric data was used for data that were non-normally distributed, while a parametric technique (*T*-test for independent samples) was used for variables that were normally distributed. The correlation between the evaluated outcomes and the clinical parameters (SSS, timed up and go and FES-I) was verified through Pearson’s product-moment correlation coefficient. Effect size (ES) was calculated using Hedges’s g considering the following interpretation: trivial (<0.20), small (0.20, −0.49), moderate (0.50, −0.79), large (>0 .80), and too large (>1.30) (Rosenthal, 1996). Hedges’s g is a variation of Cohen’s d correcting probable biases attributed to small sample size (Grissom and Kim, 2005). A significance level of ɑ = 0.05 was adopted and all data were analyzed in the Statistical Package for the Social Science (SPSS), version 22.0.

## Results

The study included 33 individuals divided into two groups: AK-R (*n* = 16) and HYP (*n* = 17). The sample characterization data are shown in Table 2. The groups showed statistically significant differences for the characterization variables referring to disease staging (Hoehn and Yahr scale, *p* = 0.031) and motor symptoms (UPDRS-III, *p* = 0.029).

**TABLE 2:** Sample characterization of akinetic-rigid and hyperkinetic groups.

Variable	Akinetic-rigid (*n* = 16)	Hyperkinetic (*n* = 17)	*p-*value
Age (years)	61.4 ± 10.2	69.2 ± 9.1	0.550
Gender (female/male)	4 / 12	5 / 12	0.452
Body mass (kg)	80.5 ± 10.6	74.7 ± 15.7	0.221
Height (m)	1.72 ± 0.10	1.67 ± 0.10	0.172
BMI (kg/m^2^)	27.1 ± 3.6	26.5 ± 4.2	0.670
Time of diagnosis (years)	6.9 ± 4.2	6.2 ± 4.2	0.637
H and Y scale (points)	2.0 ± 1.0	1.5 ± 0.5	0.031*
UPDRS-III (score)	16.1 ± 3.7	10.7 ± 3.8	0.029*
MoCA (score)	24.0 ± 0.7	24.8 ± 0.8	0.234
TUG test (s)	11.33 ± 0.81	10.26 ± 0.38	0.195
Self-selected speed (m/s)	1.40 ± 0.09	1.41 ± 0.06	0.957
FES-I (score)	22 ± 2.9	29 ± 2.1	0.665
LEDD	771.8 ± 517.1	429.4 ± 176.8	0.016*

*BMI, body mass index; H and Y, Hoehn and Yahr scale; UPDRS-III, Unified Parkinson’s Disease Rating Scale part III; MoCA, montreal cognitive assessment; TUG, timed up and go test; FES-I, Falls Efficacy Scale International; LEDD, levodopa equivalent daily dose.*

*Indicates statistically significant difference (*p* < 0.05).

### Gait Initiation

**FIGURE 2:**
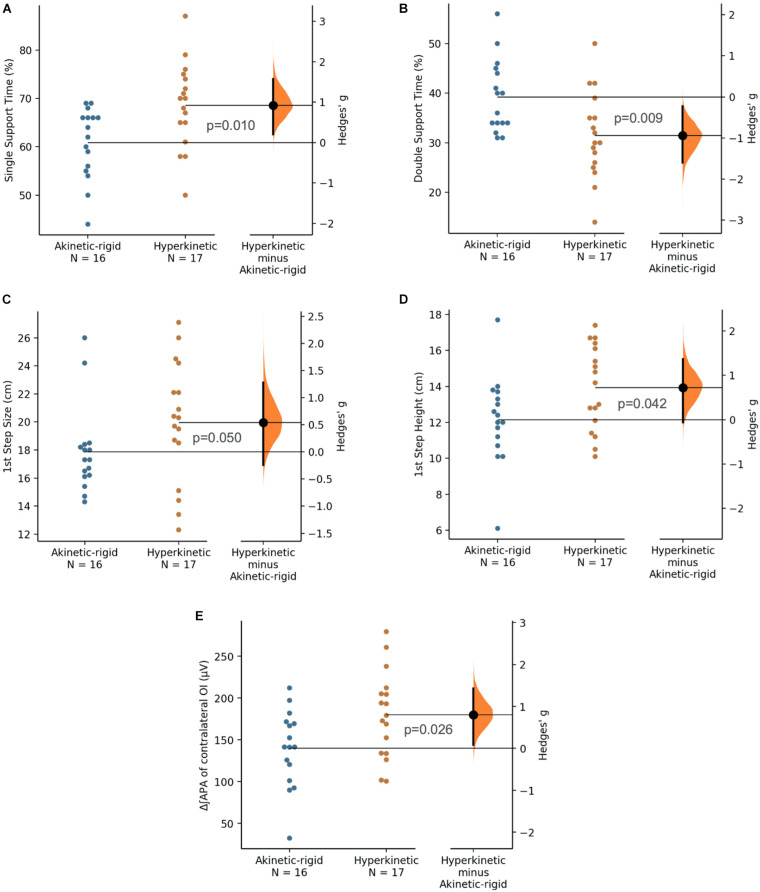
Single support time (% of stride time, **A**), double support time (% of stride time, **B**), first step size (cm, **C**), first step height (cm, **D**), and electromyographic activation of ipsilateral obliquus internus during anticipatory postural adjustment phase (μV, **E**) for akinetic-rigid (blue circles) and hyperkinetic (orange circles) groups in the gait initiation test. The Hedges’ g between akinetic-rigid and hyperkinetic is shown in the Gardner-Altman estimation plot. Both groups are plotted on the left axes; the mean difference is plotted on floating axes on the right as a bootstrap sampling distribution. The mean difference is depicted as a black circle; 95% confidence interval is indicated by the ends of the vertical error bar.

The kinematic data of gait initiation test are described in Table 3. The AK-R group had a longer double [*p* = 0.009 (ES: 0.93)] and single [*p* = 0.010 (ES: 0.92)] support, smaller size [*p* = 0.050 (ES: 0.54)] and height of the first step than in HYP group [*p* = 0.042 (ES: 0.71)] (Figure 2). The total stride time and angular kinematics of the hip, knee and ankle did not show statistical differences. Moreover, a small negative correlation (*R* = −0.432, *p* =0.012) was found between the range of ankle motion and the timed up and go test. No other correlation was found between the kinematic outcomes and the clinical-functional parameters evaluated.

**TABLE 3:** Mean, standard error, statistical significance, effect size (ES), correlations of anticipatory postural adjustment (APA), compensatory postural adjustment (CPA), kinematic data versus self-selected walking speed test (SSS), timed up and go test (TUG), falls efficacy scale-international (FES-I) for akinetic-rigid (AK-R), and hyperkinetic (HYP) groups during gait initiation test.

**Variables**	**AK-R**	**HYP**	***p*-value**	**ES**	**Correlations (*r* value*)***		
				
	**(*n* = 16)**	**(*n* = 17)**			**SSS**	**TUG**	**FES-I**
Stride time (s)	1.68 ± .07	1.63 ± .08	0.679	0.12	–0.190	0.312	0.221
Single support time (%)	60.8 ± 1.8	68.6 ± 2.1	0.010*	0.92	0.274	–0.152	–0.108
Double support time (%)	39.27 ± 1.8	31.41 ± 2.1	0.009*	0.93	–0.271	0.154	0.103
First step size (cm)	17.8 ± .8	19.9 ± 1.0	0.050*	0.54	–0.138	–0.015	0.058
First step height (cm)	12.1 ± 2.4	13.9 ± 2.3	0.042*	0.71	0.223	–0.069	0.002
First step width (cm)	21.2 ± 4.8	19.2 ± 4.8	0.230	0.41	–0.037	0.213	0.073
Range of hip motion (°)	26.5 ± 7.4	29.1 ± 6.0	0.221	0.36	0.107	0.120	0.100
Range of knee motion (°)	23.5 ± 6.4	23.9 ± 6.8	0.866	0.05	–0.080	0.173	–0.052
Range of ankle motion (°)	23.8 ± 2.1	26.3 ± 1.9	0.171	0.27	0.041	0.432^#^	0.293

*p < 0.05.

*^#^Correlation is significant at the 0.05 level (two-tailed).*

The kinetic data of APA and CPA in the gait initiation test are described in Table 4. There were no differences between groups in the kinetic outcomes. However, a moderate negative correlation (*R* = −0.632, *p* = 0.001) was observed between the COP mediolateral displacement during CPA and the timed up and go test. Likewise, a small positive correlation (*R* = 0.444, *p* = 0.010) was found between the vertical force peak during CPA and the self-selected speed of gait. No other correlation was found between the kinetic outcomes and the clinical-functional parameters evaluated.

**TABLE 4:** Mean, standard error, statistical significance, effect size (ES), correlations of anticipatory postural adjustment (APA), compensatory postural adjustment (CPA), kinetic data versus self-selected walking speed test (SSS), timed up and go test (TUG), falls efficacy scale-international (FES-I) for akinetic-rigid (AK-R), and hyperkinetic (HYP) groups during gait initiation test.

**Variables**	**AK-R**	**HYP**	***p*-value**	**ES**	**Correlations (*r* value*)***		
				
	**(*n* = 16)**	**(*n* = 17)**			**SSS**	**TUG**	**FES-I**
**APA**							
AP COP displacement (cm)	14.1 ± 2.0	15.2 ± 1.6	0.577	0.01	0.029	–0.024	0.014
ML COP displacement (cm)	5.4 ± 0.6	6.3 ± 1.0	0.627	0.29	0.104	–0.162	–0.251
Vertical force peak (%)	100.7 ± 0.3	101.3 ± 0.3	0.386	0.32	0.301	0.192	0.081
**CPA**							
AP COP displacement (cm)	8.9 ± 0.9	6.6 ± 0.7	0.105	0.05	0.220	0.086	0.132
ML COP displacement (cm)	12.1 ± 0.8	11.7 ± 0.8	0.659	0.15	0.267	0.632^#^	–0.141
Vertical force peak (%)	101.4 ± 0.5	100.9 ± 0.2	0.264	0.54	0.444^#^	–0.008	–0.043

*AP, anteroposterior; ML, mediolateral; COP, center of pressure.*

*^#^Correlation is significant at the 0.05 level (two-tailed).*

Electromyographic data of APA and CPA in the gait initiation test are described in Table 5. A statistical difference was observed in the ∫EMG of the OI muscle (contralateral to the step) [*p* = 0.026 (ES: 0.79)], where the AK-R group had a lower APA activity for this muscle than HYP (Figure 2). However, no other differences were found between the AK-R and HYP groups in muscle APA or CPA during gait initiation. In addition, no correlations were observed between EMG outcomes with clinical-functional parameters (SSS, timed up and go and FES-I) during the execution of this task.

**TABLE 5:** Mean, standard error, statistical significance, effect size (ES), correlations of anticipatory postural adjustment (APA), compensatory postural adjustment (CPA), electromyographic activation data versus self-selected walking speed test (SSS), timed up and go test (TUG), falls efficacy scale-international (FES-I) for akinetic-rigid (AK-R), and hyperkinetic (HYP) groups during gait initiation test.

**Variables**	**AK-R**	**HYP**	***p*-value**	**ES**	**Correlations (*r* value)**		
				
	**(*n* = 16)**	**(*n* = 17)**			**SSS**	**TUG**	**FES-I**
**APA**							

OI-ip (μV)	142.5 ± 06.9	163.7 ± 10.9	0.141	0.51	0.053	–0.128	–0.009
OI-co (μV)	139.7 ± 11.4	182.5 ± 13.0	0.026*	0.79	0.229	–0.328	–0.209
EEL-ip (μV)	165.9 ± 17.2	171.5 ± 11.9	0.564	0.04	–0.015	–0.098	–0.239
EEL-co (μV)	180.4 ± 13.7	164.1 ± 09.9	0.295	0.36	0.209	0.230	–0.081
GLM (μV)	336.1 ± 29.6	396.9 ± 49.1	0.261	0.36	0.246	–0.073	–0.107
RF (μV)	432.7 ± 38.1	548.1 ± 60.5	0.183	0.62	0.172	0.006	–0.019
BF (μV)	410.6 ± 51.7	399.0 ± 46.6	0.640	0.08	–0.182	–0.036	0.120
TA (μV)	581.3 ± 56.1	514.2 ± 50.1	0.308	0.08	–0.015	–0.089	–0.190
GAM (μV)	606.2 ± 59.5	642.5 ± 57.8	0.587	0.18	0.072	0.035	–0.112

**CPA**							

OI-ip (μV)	145.3 ± 14.2	180.0 ± 14.1	0.190	0.58	0.140	–0.129	–0.051
OI-co (μV)	169.7 ± 16.0	158.7 ± 09.5	0.776	0.20	0.085	0.041	0.089
EE-ip (μV)	166.1 ± 19.5	203.3 ± 19.8	0.079	0.49	–0.242	–0.150	–0.010
EE-co (μV)	176.0 ± 13.5	192.5 ± 13.9	0.347	0.32	–0.101	0.047	0.094
GLM (μV)	407.3 ± 53.1	421.4 ± 40.3	0.773	0.01	0.027	–0.156	–0.153
RF (μV)	504.7 ± 43.3	556.1 ± 61.6	0.470	0.28	0.238	0.121	–0.065
BF (μV)	443.9 ± 44.9	441.8 ± 47.4	0.899	0.04	–0.042	–0.216	–0.224
TA (μV)	591.7 ± 58.1	532.1 ± 55.0	0.428	0.23	–0.273	–0.131	–0.139
GAM (μV)	609.2 ± 66.9	648.0 ± 39.1	0.578	0.19	0.162	–0.048	–0.199

*OI-ip, ipsilateral obliquus internus; OI-co, contralateral obliquus internus; EEL-ip, ipsilateral erector spinae longissimus; EEL-co, contralateral erector spinae longissimus; GLM, gluteus medius; RF, rectus femuralis; BF, biceps femuralis; TA, tibialis anterior; GAM, gastrocnemius medialis; μV, microvolts.*

*p <0.05.

### Obstacle Negotiation

**FIGURE 3:**
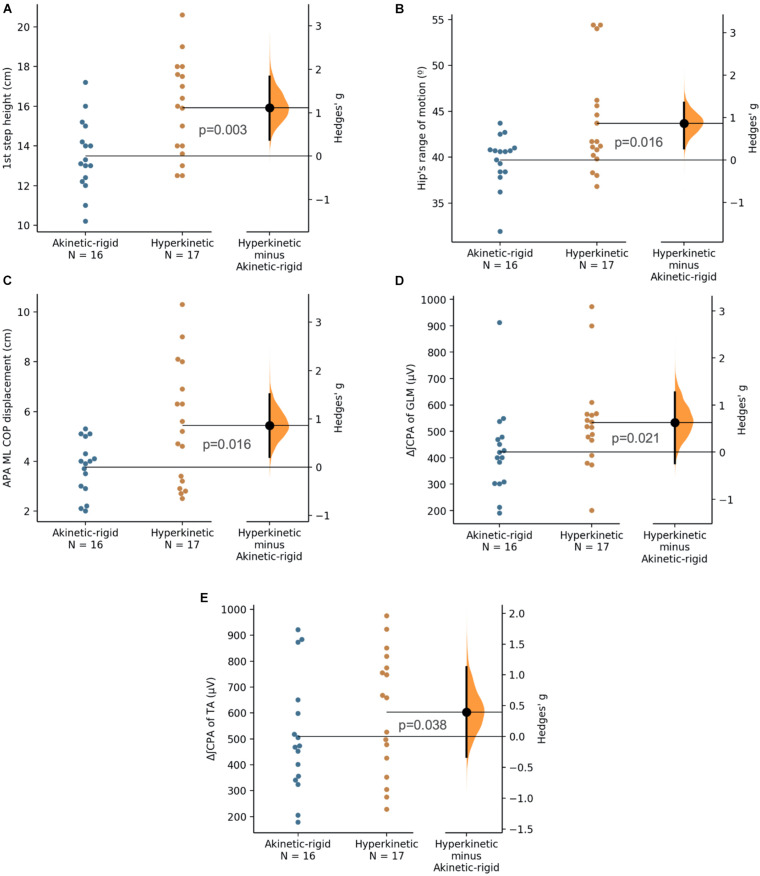
First step height (cm, **A**), range of hip motion (◯, **B**), anticipatory postural adjustment of mediolateral center of pressure displacement (cm, **C**), electromyographic activation of gluteus medius during compensatory postural adjustment phase (μV, **D**), electromyographic activation of tibialis anterior during compensatory postural adjustment phase (μV, **E**) for akinetic-rigid (blue circles) and hyperkinetic (orange circles) groups in the obstacle negotiation test. The Hedges’ g between akinetic-rigid and hyperkinetic is shown in the Gardner-Altman estimation plot. Both groups are plotted on the left axes; the mean difference is plotted on floating axes on the right as a bootstrap sampling distribution. The mean difference is depicted as a black circle. 95% confidence interval is indicated by the ends of the vertical error bar.

The kinematic data of the obstacle negotiation test are described in Table 6. The AK-R group had a height of the first step [*p* = 0.003 (ES: 1.11)] and hip’s range of motion [*p* = 0.016 (ES: 0.86)] smaller than the HYP group (Figure 3). The other variables did not show statistical differences. In addition, a small negative correlation (*R* = −0.452, *p* = 0.008) was found between the first step height and the FES-I score. Also, a small positive correlation was observed between FES-I and the range of ankle motion (*R* = 0.365, *p* = 0.037). No other correlation was found between the kinematic outcomes and the clinical-functional parameters evaluated.

**TABLE 6:** Mean, standard error, statistical significance, effect size (ES), correlations of anticipatory postural adjustment (APA), compensatory postural adjustment (CPA), kinematic data versus self-selected walking speed test (SSS), timed up and go test (TUG), falls efficacy scale-international (FES-I) for akinetic-rigid (AK-R), and hyperkinetic (HYP) groups during obstacle negotiation test.

**Variables**	**AK-R**	**HYP**	***p*-value**	**ES**	**Correlations (*r* value*)***		
				
	**(*n* = 16)**	**(*n* = 17)**			**SSS**	**TUG**	**FES-I**
Stride time (s)	2.04 ± 0.11	1.93 ± 0.97	0.201	0.43	–0.176	0.135	0.205
Single support time (%)	63.3 ± 2.2	67.3 ± 2.3	0.160	0.27	0.065	–0.104	–0.252
Double support time (%)	36.7 ± 2.2	32.7 ± 2.3	0.160	0.27	–0.065	0.104	0.252
First step size (cm)	16.2 ± 4.4	16.8 ± 3.7	0.732	0.11	0.300	–0.253	–0.058
First step height (cm)	13.5 ± 1.8	15.9 ± 2.4	0.003*	1.11	–0.070	–0.132	−0.452^#^
First step width (cm)	21.8 ± 1.4	21.9 ± 0.9	0.105	0.01	–0.040	0.120	–0.017
Range of hip motion (°)	39.6 ± 0.7	43.6 ± 1.4	0.016*	0.86	–0.131	0.031	0.145
Range of knee motion (°)	47.2 ± 2.3	51.6 ± 3.0	0.262	0.39	–0.086	0.168	0.146
Range of ankle motion (°)	24.2 ± 8.2	24.8 ± 7.5	0.835	0.07	0.007	0.217	0.365^#^

*p < 0.05.

*^#^Correlation is significant at the.05 level (two-tailed).*

The kinetic data of APA and CPA in the obstacle negotiation test are described in Table 7. The AK-R group presented APA with less mediolateral COP displacement [*p* = 0.016 (ES: 0.86) than the HYP group (Figure 3). There were no significant differences between groups for other kinetic variables during APA and CPA in the gait initiation test. Besides that, a small negative correlation (*R* = −0.358, *p* = 0.041) was observed between the mediolateral displacement of the COP during CPA and FES-I score.

**TABLE 7:** Mean, standard error, statistical significance, effect size (ES), correlations of anticipatory postural adjustment (APA), compensatory postural adjustment (CPA), kinetic data versus self-selected walking speed test (SSS), timed up and go test (TUG), falls efficacy scale-international (FES-I) for akinetic-rigid (AK-R), and hyperkinetic (HYP) groups during obstacle negotiation test.

	**AK-R**	**HYP**	***p*-value**	**ES**	**Correlations (*r* value*)***		
				
	**(*n* = 16)**	**(*n* = 17)**			**SSS**	**TUG**	**FES-I**
**APA**							

AP COP displacement (cm)	12.9 ± 0.7	11.6 ± 0.6	0.206	0.44	–0.085	–0.152	0.243
ML COP displacement (cm)	3.7 ± 0.3	5.4 ± 0.5	0.016*	0.86	–0.165	0.161	0.230
Vertical Force Peak (%)	100.7 ± 0.3	101.3 ± 0.3	0.449	0.03	0.331	0.037	–0.244

**CPA**							

AP COP displacement (cm)	5.7 ± 0.4	5.2 ± 0.4	0.430	0.02	–0.106	0.030	–0.067
ML COP displacement (cm)	13.4 ± 0.6	13.5 ± 0.6	0.924	0.03	0.067	–0.190	−0.358^#^
Vertical Force Peak (%)	101.4 ± 0.5	100.9 ± 0.2	0.517	0.01	0.169	–0.290	–0.308

*AP, anteroposterior; ML, mediolateral; COP, center of pressure.*

*p < 0.05.

*^#^Correlation is significant at the 0.05 level (two-tailed).*

Electromyographic data of APA and CPA in the obstacle negotiation test are described in Table 8. No significant difference was observed between muscle activity in APA time of the two groups, as for CPA time, when less activity of GLM [*p* = 0.021 (ES: 0.63)] and TA [*p* = 0.038 (ES: 0.39)] in the AK-R group (Figure 3). The other muscles showed no statistical difference in CPA between the two groups.

**TABLE 8:** Mean, standard error, statistical significance, effect size (ES), correlations of anticipatory postural adjustment (APA), compensatory postural adjustment (CPA), electromyographic activation data versus self-selected walking speed test (SSS), timed up and go test (TUG), falls efficacy scale-international (FES-I) for akinetic-rigid (AK-R), and hyperkinetic (HYP) groups during obstacle negotiation test.

	**AK-R**	**HYP**	***p-*value**	**ES**	**Correlations (*r* value)**		
				
	**(*n* = 16)**	**(*n* = 17)**			**SSS**	**TUG**	**FES-I**
**APA**							

OI-ip (μV)	144.3 ± 13.3	17.6 ± 18.4	0.183	0.46	–0.019	0.030	0.210
OI-co (μV)	185.3 ± 22.9	168.8 ± 14.7	0.589	0.20	–0.077	0.221	0.162
EEL-ip (μV)	172.0 ± 11.3	185.0 ± 23.9	0.692	0.17	–0.206	0.353^#^	–0.029
EEL-co (μV)	199.1 ± 23.9	179.3 ± 09.8	0.729	0.26	0.050	0.044	–0.082
GLM (μV)	347.7 ± 29.3	374.5 ± 30.7	0.423	0.21	0.214	–0.192	0.754
RF (μV)	495.1 ± 64.1	580.1 ± 40.6	0.265	0.38	–0.073	0.486^#^	0.064
BF (μV)	396.5 ± 55.5	418.8 ± 38.3	0.221	0.11	–0.210	0.452^#^	0.151
TA (μV)	643.4 ± 53.1	632.0 ± 43.9	0.870	0.05	–0.090	0.034	–0.019
GAM (μV)	609.4 ± 54.1	680.0 ± 53.3	0.360	0.31	0.037	–0.146	–0.235

***CPA***							

OI-ip (μV)	161.0 ± 15.1	177.8 ± 15.0	0.614	0.26	0.001	0.059	0.198
OI-co (μV)	192.9 ± 20.3	182.0 ± 15.1	0.668	0.14	–0.173	–0.136	0.014
EE-ip (μV)	162.6 ± 15.9	177.7 ± 12.7	0.140	0.39	–0.073	–0.011	–0.112
EE-co (μV)	171.6 ± 17.6	182.8 ± 13.6	0.197	0.17	0.124	0.074	0.094
GLM (μV)	421.1 ± 41.7	533.8 ± 43.8	0.021*	0.63	–0.236	0.138	0.093
RF (μV)	588.0 ± 56.4	648.3 ± 41.4	0.392	0.29	–0.188	0.158	0.391^#^
BF (μV)	440.0 ± 41.0	481.8 ± 39.6	0.470	0.24	–0.114	–0.206	–0.030
TA (μV)	509.1 ± 56.9	603.2 ± 57.3	0.038*	0.39	–0.202	0.017	–0.001
GAM (μV)	608.2 ± 64.4	617.9 ± 43.1	0.901	0.04	–0.010	–0.180	–0.067

*OI-ip, ipsilateral obliquus internus; OI-co, contralateral obliquus internus; EEL-ip, ipsilateral erector spinae longissimus; EEL-co, contralateral erector spinae longissimus; GLM, gluteus medius; RF, rectus femoris; BF, biceps femoris; TA, tibialis anterior; GAM, gastrocnemius medialis; μV, microvolts.*

*p < 0.05.

*^#^Correlation is significant at the 0.05 level (two-tailed).*

## Discussion

We investigated the differences in postural adjustments (APA and CPA) and biomechanical parameters during gait initiation and obstacle negotiation tasks between akinetic-rigid and hyperkinetic PD. Furthermore, we correlated these parameters with clinic functional parameters. We confirmed the study’s hypothesis since AK-R subtype exhibited less effective APA and CPA, reflected in lower EMG signal from the stabilizing muscles and a smaller displacement of the COP. Also, AK-R people performed the tasks with shorter step size, step height, and range of limb joints motion.

Studies indicate clear clinical evidence that the PD subtypes have different clinical courses (Jankovic and Kapadia, 2001; Rajput et al., 2009). Hyperkinetic patients tend to have slower disease progression and less cognitive decline than akinetic rigid patients. This response may be related to different neuropathological findings and biochemical abnormalities between PD subtypes (Jellinger, 1999; Marras et al., 2002; Rajput et al., 2009). In fact, in our study, although the sample was homogeneous for age, gender, body measurements, time of diagnosis, cognitive aspects, and performance in clinical-functional tests, there was a difference in the H and Y score and the UPDRS-III score, both higher for the AK-R group, suggesting that patients with this subtype, tend to show a faster progression of motor impairment. According to our results, these differences are manifested in the balance and locomotion during complex tasks as gait initiation and negotiating obstacles.

Previous studies (Mellroy and Maki, 1999; Winter et al., 2003) reported that the center of mass displacement toward the support leg by individuals with PD il lower than in age-matched healthy elderly. Our results demonstrated a smaller mediolateral center of pressure displacement before the obstacle negotiation in the AK-R group. PD patients generally show a reduced APA effectiveness (Mancini et al., 2009; Rocchi et al., 2012; Delval et al., 2014) and a reduced ability to adjust APA and CPA in situations with greater balance disturbance or insecurity sensation, such as overcoming an obstacle (Vitório et al., 2010). This mechanism can be further impaired in PD exhibiting rigidity, bradykinesia or freezing episodes. Considering that the effectiveness of postural adjustments involves inhibitions and activation of the postural musculature, plastic spasticity, occurring in PD, added to the fear of falling, affecting the effectiveness of motor responses that recover or prevent the significant center of mass imbalances, increasing the risk of falls. Although we have not found other studies comparing the APA and CPA of individuals with AK-R and HYP PD, Schlenstedt et al. (2018) investigated APA at the beginning of the gait of patients with PD with and without freezing of gait. They noted that people with PD with a history of freezing of gait (“off” medication status) had lower mediolateral and anteroposterior APA compared to individuals without a history of freezing of gait, especially in a dual-task situation. Other studies did not find differences in APA displacements when comparing PD with and without freezing of gait (Delval et al., 2014; Plate et al., 2016), but in the “on” medication state, which may justify different findings.

Although there was no statistical difference between AK-R and HYP in the gait initiation test, some correlations were found with the clinical-functional parameters evaluated. A negative correlation was observed between the APA mediolateral displacement of the COP and the timed up and go test, so that the smaller the COP displacement, the longer the time needed to perform the timed up and go test. This test assesses functional mobility and has been used to assess motor performance and predict the risk of falls (Bischoff et al., 2003; Guimarães et al., 2013). During the execution of a task that includes getting up from a chair, starting to walk and going around an obstacle, it is essential to have an effective postural balance and postural adjustments that involve controlling the center of mass along with the steps. Furthermore, a positive correlation was found between peak vertical force and self-selected gait speed, which corroborates studies that observed deficits in forward propulsion and gait speed in individuals with PD as the disease progresses (Winogrodzka et al., 2005). A large APA is associated with better motor performance during self-started gait, resulting in greater gait speed. This response is related to effective postural control and the production of propulsive forces, considering that the forces caused by the APA seem to help in the acceleration of the center of mass forward (Schlenstedt et al., 2018). Thus, our results indicate that the analysis of PD subtypes is necessary to understand better and clinical management of these outcomes. Furthermore, a lower mediolateral displacement of the COP may be related to rigidity and less tissue and joint compliance in the retro and midfoot (Saghazadeh et al., 2014), knee, hip (Tateuchi et al., 2011) and even in the physiological trendelenburg – due to the single-leg support and the eccentric action of the hip abductors-, or even by the co-contraction of hip and pelvis muscles (Delafontaine et al., 2019).

The kinematic results showed greater differences between akinetic-rigid and hyperkinetic. During gait initiation, the AK-R group had a shorter period of single support and a longer period of double support than the HYP group. In addition, the results indicate smaller step size and shorter step height for the AK-R group. Likewise, we found statistical differences during the negotiation of obstacles in the step height and range of hip motion. Although these variables have not been compared between AK-R and HYP PD in other studies, Okada et al. (2011) found that people with PD with freezing of gait had longer periods of double support during the first three steps than those without freezing of gait. These findings corroborate with other studies that found an increase in the time of double support and a reduction in the step size in PD, especially when associated with freezing, the advance of motor symptoms or “off” state of the medication (Hausdorff et al., 2003; Schaafsma et al., 2003; Iansek et al., 2006). Thus, differences in single and double support periods may suggest that people with akinetic-rigid PD have less dynamic stability than hyperkinetic.

Akinetic-rigid group exhibited a lower step height, accompanied by a lower range of hip motion during the obstacle negotiation. The step height is a determining factor in the execution of this task (Vitório et al., 2010; Kim et al., 2014). People who have a foot lift close to the height of the obstacle are more prone to falls and injuries. Our results suggest a negative correlation between the step height during obstacle negotiation and the FES-I score, so that the lower the step height, the higher the score tends to be and, consequently, the greater the fear of falling. In this sense, Vitório et al. (2010) investigated the locomotor responses of people with PD during obstacle negotiation. Although they have compared people with PD and healthy older adults, differences in size, height, and stride time were observed, with greater deficits in the population with neurological impairment in line with our findings.

During gait initiation, a difference was observed in the EMG of the OI muscle (contralateral to the step), where the AK-R group had a lower ∫EMG activity in OI contralateral muscle in APA time than HYP. There were no differences between the APA of the other muscles or CPA. In the obstacle negotiation, the AK-R group had lower ∫EMG activity in CPA time of the GLM and TA muscles. In tasks in which it is necessary to change from a static to a dynamic situation, stabilizing muscles have a fundamental role in controlling balance during the execution. Studies show that OI seems to be activated early in APA situations (Hodges and Richardson, 1997; Morris and Alisson, 2006). The deep abdominal musculature seems to be directly involved in motor responses to prevent further displacements of the center of mass. Subjects with low back pain and post-stroke have lower activities of the deep abdominal muscles during postural adjustments (Dickstein et al., 2004; Jacobs et al., 2008). In addition, the deep abdominal musculature appears to be involved in dystonias, possibly related to changes in postural tone such as camptocormia and Pisa’s syndrome (Reichel et al., 2001; Tassorelli et al., 2012). Thus, the difference found in the contralateral OI activity between the subgroups HYP and AK-R may be related to the impairment of structures related to posture and balance, such as those studied here. Moreover, some authors suggest that people with PD have deficient postural adjustments of stabilizing muscles, including deep abdominals, hip abductors and anterior tibialis, when compared to healthy groups (Gantchev et al., 1996).

In healthy subjects, a bilateral reduction in the tonic activity of the gastrocnemius is described, accompanied by a bilateral increase in the phasic activity of the tibialis anterior to the first step. This action occurs in order to control the displacement of the center of mass backward and toward the supporting member while the executing member progresses to take off (Assaiante et al., 2000; Mickelborough et al., 2004; Di Giulio et al., 2009). Also, PD patients are known to generate insufficient dorsiflexion torque due to inappropriate tibialis anterior activation during the initiation of gait (Gantchev et al., 1996; Halliday et al., 1998). These changes seem to be more evident as the disease progresses and the presence of motor symptoms such as freezing. The decrease in step size during the beginning of gait (Morris et al., 1996; Jacobs et al., 2009) may reflect some of these abnormalities in the postural phase. Then, a lower phasic activity of the TA may mean insufficient activity to contain the displacement of the posterior center of mass and the consequent tendency to fall backward. Thus, in our study, the lower TA activity of the AK-R group may incur a greater deficit in balance control at the first step, when compared to the HYP group.

Hip abductors, on the other hand, play a fundamental role in controlling the lateral movement of the center of mass and the lateral loading/unloading mechanism during the support phase (Winter et al., 1993, 1996) and gait (Mickelborough et al., 2004). During the gait initiation, postural adjustments in the frontal plane accelerate the center of mass toward the support side, allowing the oscillating foot to be raised (Lyon and Day, 1997), with the GLM having an important action at this moment (Winter et al., 1996; Kirker et al., 2000). Thus, considering our results, the lower GLM activity of the AK-R group during obstacle negotiation may incur a greater deficit in postural control and task performance, compared to HYP group.

When gait initiation is associated with a factor that increases the challenge of maintaining balance, such as the presence of an obstacle, these changes may be more evident. The negotiation of the obstacle requires greater elevation of the executing limb and greater control of the single-leg support, and there may be an even greater exacerbation of symptoms due to psychosocial factors, such as fear of falling (Vitório et al., 2010). In our study, although no significant difference was observed at the gait initiation other than the action of OI, less compensatory actions of the GLM and TA muscles were found during the obstacle negotiation, a situation in which there is a greater magnitude of the postural disorder, as mentioned. Likewise, although there were no differences in kinetic data during the start of the gait, a smaller mediolateral displacement of the COP was observed before the obstacle negotiation for the AK-R group. This reduced ability to modulate the ML displacement of the COP may be due to changes in the strength of the proximal musculature, mainly of the hip muscles (Kim et al., 2014). This explanation corroborates with our results, which demonstrate a smaller ML COP displacement associated with a lower GLM action before the obstacle negotiation for the AK-R group, which also had a worse score on motor symptoms (UPDRS-III) and staging (Hoehn and Yahr).

Our results indicate that the understanding of the motor behavior of subjects with PD during activities involving balance and locomotion should be explored in more detail in each subgroup. Understanding the motor behavior of PD subgroups can be an important key to clinical management and exercise prescription, which should seek to minimize the specific impacts of each subgroup of this very common and crippling neurodegenerative disease.

A potential limitation of our study is that all participants were evaluated only in the “on” phase of the medication, so that symptoms and motor impairments may have been mitigated. In addition, the participants were classified between 1 and 3 according to the Hoehn and Yahr scale (mild to moderate PD). Thus, future studies should investigate these parameters in more homogeneous groups. Also, further studies are needed to assess postural automatisms and biomechanics during gait initiation and obstacle negotiation in participants with akinetic-rigid and hyperkinetic PD in the inactive phase of medication, with an even more evident difference. Still, we suggest new studies that investigate postural automatisms and the performance of other daily tasks comparing subjects with different clinical manifestations of symptoms and clinical trials that aim to evaluate the effects of different interventions and stimuli on the evaluated parameters (Monteiro et al., 2017; Zanardi et al., 2019). In addition, we suggest cross-sectional studies that investigate the acute effects of interventions and stimuli on the motor behavior of this population.

## Conclusion

We demonstrated that individuals with AK-R PD have impaired APA and CPA during gait initiation and obstacle negotiation tasks when compared to the HYP PD group. Shorter oscillation and lower muscle activation in postural muscles seem to affect the gait biomechanical parameters and functional mobility in PD. These results help to understand the differences in motor control presented according to the clinical manifestation of people with PD, suggesting that the subtype of the disease should be considered in clinical practice.

## Data Availability Statement

The datasets presented in this study can be found in online repositories. The names of the repository/repositories and accession number(s) can be found below: doi: 10.6084/m9.figshare.14759037.v1.

## Ethics Statement

The studies involving human participants were reviewed and approved by Universidade Federal do Rio Grande do Sul. The patients/participants provided their written informed consent to participate in this study.

## Author Contributions

MC, LP-T, FM, and AH conceived of the study and designed the experiments. LP-T obtained the funding. MC, AZ, AI-M, LA, LP-T, and FM carried out the analysis, interpreted the statistical results, and drafted the manuscript. MC, AZ, and AI-M collected the data. All authors contributed to the manuscript writing, read, and approved the final manuscript.

## Conflict of Interest

The authors declare that the research was conducted in the absence of any commercial or financial relationships that could be construed as a potential conflict of interest.

## Publisher’s Note

All claims expressed in this article are solely those of the authors and do not necessarily represent those of their affiliated organizations, or those of the publisher, the editors and the reviewers. Any product that may be evaluated in this article, or claim that may be made by its manufacturer, is not guaranteed or endorsed by the publisher.
